# Time to disease-related pain and first opioid use in patients with metastatic castration-resistant prostate cancer treated with sipuleucel-T

**DOI:** 10.1038/pcan.2014.21

**Published:** 2014-06-24

**Authors:** E J Small, C S Higano, P W Kantoff, J B Whitmore, M W Frohlich, D P Petrylak

**Affiliations:** 1 Helen Diller Family Comprehensive Cancer Center, University of California at San Francisco, San Francisco, CA, USA; 2 Departments of Oncology and Urology, School of Medicine, University of Washington, Seattle, WA, USA; 3 Dana-Farber Cancer Institute, Harvard Medical School, Boston, MA, USA; 4 Dendreon Corporation, Seattle, WA, USA; 5 Smilow Cancer Center, Yale University, New Haven, CT, USA

## Abstract

**Background:**

Sipuleucel-T has demonstrated improved overall survival in patients with metastatic castration-resistant prostate cancer (mCRPC). This analysis examined the effect of sipuleucel-T on time to disease-related pain (TDRP) and time to first use of opioid analgesics (TFOA) in mCRPC using data pooled from three randomized phase III studies in men with asymptomatic or minimally symptomatic mCRPC (D9901 (NCT00005947), D9902A (NCT01133704), D9902B (IMPACT; NCT00065442)).

**Methods:**

Four-hundred and twenty-eight asymptomatic patients were analyzed for TDRP; 737 patients were analyzed for TFOA. Pain status was collected using logs adjudicated by blinded, independent reviewers. Opioid use for cancer-related pain was identified from medically reviewed reports of concomitant medication. Disease-related pain was defined as pain post enrollment. TDRP and TFOA were analyzed using the Kaplan–Meier method and Cox regression.

**Results:**

Treatment with sipuleucel-T was not associated with a significant difference in TDRP (hazard ratio (HR)=0.819; 95% confidence interval (CI): 0.616–1.089; *P=*0.170; median TDRP 5.6 months for sipuleucel-T and 5.3 months for control, respectively), although 39.3% of sipuleucel-T-treated patients and 18.9% of control patients were pain-free at 12 months. However, there was a significant delay in TFOA with sipuleucel-T (HR=0.755; 95% CI: 0.579–0.985; *P=*0.038). Median TFOA for sipuleucel-T was 12.6, and 9.7 months for control, with 50.6% and 43.1% opioid-free at 12 months, respectively. Kaplan–Meier curves for both end points began to diverge at 6 months.

**Conclusions:**

Sipuleucel-T was associated with longer TFOA but not significantly longer TDRP. Both end points demonstrated evidence of a delayed treatment effect, consistent with an active immunotherapy.

## Introduction

The presence of pain in patients with metastatic castration-resistant prostate cancer (mCRPC) is an adverse prognostic marker for survival.^1, 2^ However, the delay in time to disease-related pain (TDRP) or first use of opioid analgesics (TFOA) provided by a therapeutic agent, while clearly important clinical attributes, have not been validated as prognostic or predictive markers. Data regarding these attributes were prospectively collected in three clinical trials evaluating sipuleucel-T (Provenge; Dendreon Corporation, Seattle, WA, USA), and provided an opportunity to explore these pain-related end points.

Sipuleucel-T is an autologous cellular immunotherapy designed to stimulate an immune response against the prostate tumor antigen prostatic acid phosphatase.^3, 4^ The efficacy and safety of sipuleucel-T in asymptomatic or minimally symptomatic mCRPC have been investigated in three phase III trials. D9901 (NCT00005947) demonstrated a significant reduction in the risk of death with sipuleucel-T versus control.^4^ In D9902A (NCT01133704) a trend towards improved survival was observed despite early discontinuation of enrollment.^5^ The pivotal IMPACT trial (D9902B (NCT00065442)) demonstrated a significant 22% reduction in the risk of death (*P*=0.03) with sipuleucel-T versus control and a 4.1-month improvement in median overall survival (OS).^6^

In all three trials, TFOA and TDRP data were prospectively collected in a blinded fashion and TDRP was a prospectively defined end point.^4, 5, 6^ Given the similar study designs, a *post hoc* pooled analysis was undertaken to increase the sample size and improve the precision of treatment-effect estimates. TDRP was analyzed for all patients known to be asymptomatic at enrollment. TFOA was evaluated for all patients, irrespective of their symptomatic status at enrollment.

## Materials and methods

### Study design

Table 1 summarizes the designs of the three phase III sipuleucel-T trials in mCRPC.^4, 5, 6^ All trials enrolled patients with Eastern Cooperative Oncology Group performance status (ECOG PS) 0 or 1, average weekly pain score <4 (10-point visual analog scale) at registration, and no visceral metastases. D9901/D9902A recruited only asymptomatic patients but with any Gleason score (GS). IMPACT initially enrolled only asymptomatic patients with GS ⩽7, but was amended following data review from D9901 and D9902A to include patients with any GS and minimally symptomatic disease (symptoms not requiring treatment with opioid analgesics within 21 days before registration). Pain correlating with a site of metastatic disease was allowed provided it met the above pain score criterion. TDRP was a secondary end point in D9901/D9902A, and data were intended to be pooled. Originally, in IMPACT, TDRP was a co-primary end point and TFOA was a secondary end point; both end points were removed as the protocol amendment allowed symptomatic patients and TDRP became irrelevant.

The intent-to-treat population for the TDRP analysis (*n*=428) included all randomized patients in D9901 (*n*=127) and D9902A (*n*=98), and asymptomatic patients from IMPACT randomized prior to the protocol amendment (*n*=203). The intent-to-treat population for the TFOA analysis (*n*=737) included all randomized patients in D9901/D9902A/IMPACT.

Patients were randomized 2:1 to receive sipuleucel-T or control^4, 5, 6^ and were followed until disease progression (PD). After central confirmation of PD, patients were treated at the physician's discretion. Control patients could join an open-label salvage protocol and receive APC8015F, a product manufactured similarly to sipuleucel-T but from the remaining cells cryopreserved when the control was prepared.

Studies were conducted in compliance with US Food and Drug Administration and Good Clinical Practice guidelines, and met local Institutional Review Board approvals. Written informed consent was obtained from all patients.

### Disease progression

For D9901/D9902A, the PD end point included progression of measurable or evaluable disease (for example, lesions on bone scans), spinal-cord compression or pathological fracture at known disease site(s), developing a requirement for radiotherapy, or other clinically significant disease-specific events, including disease-related pain (DRP) or other symptoms.^4, 5^ For IMPACT, the PD end point included only objective PD, defined as first progression of measurable or evaluable disease confirmed by an independent, blinded review committee.^6^ No study used serum PSA increase to measure PD.

### TDRP and TFOA

In all studies, pain logs were collected and adjudicated by blinded, independent reviewers. DRP was defined as pain with a quality and consistency of cancer-related pain that occurred after study enrollment and anatomically correlated with the site(s) of radiographically demonstrated disease. In D9901/D9902A, onset of DRP and opioid analgesic use (OAU) were documented using a weekly log. Patients rated their pain from 0 (no pain) to 10 (pain as bad as can be imagined) for three questions from the Brief Pain Inventory.^7^ DRP was considered present if the patient had unusual pain or a ⩾3-point increase in intensity at a baseline pain site. In IMPACT, pain and medication use were recorded with weekly patient-completed logs and monthly investigator-completed logs. DRP was considered present if pain was reported at a new site and the average intensity was ⩾2, and/or if existing pain intensity increased by ⩾2 points from screening. Date of pain onset was identified via a blinded, dual review of pain logs and a ‘DRP' worksheet that was completed when DRP was identified.

In D9901/D9902A, pain and analgesic-use status was collected until DRP occurred or for 4 weeks following PD, whichever happened first. For patients without DRP by 4 weeks after PD, data were censored at the last available pain evaluation. In IMPACT, pain and medication status was collected until DRP occurred, regardless of the time since PD. Following protocol amendment, completion of pain logs was discontinued and data were censored at the last pain evaluation for patients who had not reached the pain end point.

A blinded medical review identified OAU by preferred drug names in the coded concomitant medication information on case report forms, and excluded OAU unrelated to cancer pain. OAU was excluded for procedures, rigors related to infusions, lasting ⩽2 days, or clearly unrelated to cancer.

TDRP and TFOA were defined as the time from randomization to first identification of DRP or OAU, respectively.

### Statistical considerations

The individual studies were underpowered for the TDRP and TFOA end points. Accordingly, statistical analysis was performed on pooled data. The statistical analysis plan for D9901/D9902A prespecified that TDRP data were to be pooled; these combined studies had 80% power to detect a hazard ratio (HR) of 0.52 at the two-sided α=0.05 level, provided that 80 pain events were observed. Prior to amendment, IMPACT had 90% power to detect an HR of 0.562 at the two-sided α=0.01 level, provided that 193 pain events were observed. Based on the 212 DRP events observed across the three studies, a *post hoc* power analysis indicated there was 80% power to detect an HR of 0.67 at the two-sided α=0.05 level. Similarly, based on 240 OAU events, a *post hoc* power analysis indicated there was 80% power to detect an HR of 0.68 at the two-sided α=0.05 level.

TDRP and TFOA were summarized using the Kaplan–Meier method. Two-tailed *P*-values, HRs and 95% confidence intervals (CIs) were derived from a Cox regression model, adjusted for baseline PSA and lactate dehydrogenase (LDH) (both log-transformed). Integrated analyses were stratified by study.

*Post hoc* exploratory analyses were conducted using two separate Cox models, stratified by study and employing stepwise selection, to identify independent predictors of TDRP or TFOA from the following: previous chemotherapy, hemoglobin, PSA, alkaline phosphatase, LDH, age, number of bone metastases, bisphosphonate use, GS, ECOG PS, weight, time from diagnosis to randomization, previous primary radiotherapy, race (Caucasian versus others), presence of soft tissue disease, and prior radical prostatectomy. Treatment arm (sipuleucel-T or control) was included in the resulting models to assess treatment effect after adjustment.

## Results

### Patient characteristics and disposition

**Figure 1:**
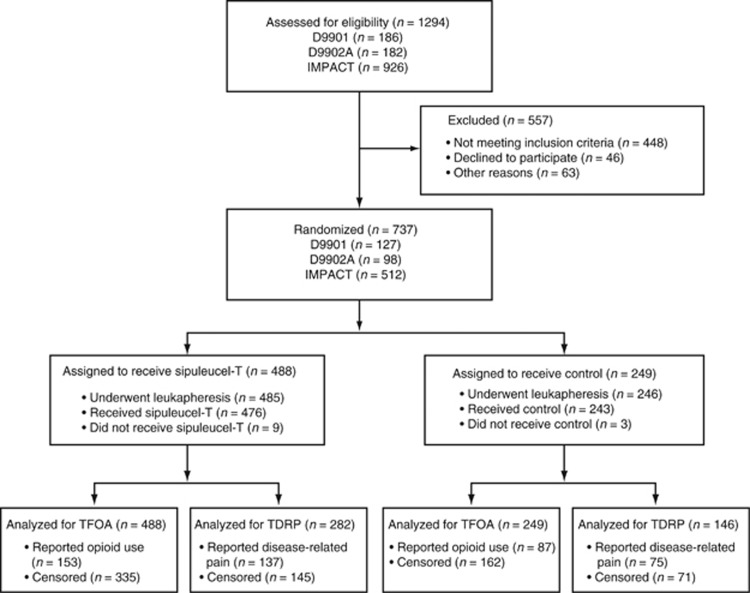
Patient enrollment and outcomes. TDRP, time to disease-related pain; TFOA, time to first use of opioid analgesics.

Figure 1 summarizes the disposition of patients included in the TFOA and TDRP analyses. Patient characteristics were well balanced between the arms (Table 2), although more sipuleucel-T than control patients in the TDRP population had received previous primary radiotherapy; there was no difference in prior radiotherapy between the two arms for the TFOA analysis. Fewer sipuleucel-T than control patients in both the TDRP and TFOA populations had soft-tissue disease. A prognostic model incorporating baseline PSA, LDH, alkaline phosphatase, hemoglobin, ECOG PS, GS and the presence of visceral disease^8^ predicted that survival for the sipuleucel-T and control arms, respectively, was comparable for the TDRP (21.6 and 21.5 months) and TFOA (20.1 and 20.1 months) populations. Of control patients, 99 (67.8%) in the TDRP population and 165 (66.3%) in the TFOA population subsequently received APC8015F following PD.

### TDRP

PD was documented in most of the 428 patients analyzed for TDRP, with 243/282 (86.2%) sipuleucel-T and 132/146 (90.4%) control patients progressing (Table 3a); DRP was documented in 137/282 (48.6%) and 75/146 (51.4%) patients, respectively (Table 3a). Censoring rates for TDRP were high in both the sipuleucel-T (51.4% 145/282) and control (48.6% 71/146) arms because pain logs were discontinued post-amendment in IMPACT and determination of pain status was discontinued 4 weeks following PD in D9901/D9902A, with PD occurring before pain in 57.3% (129/225) of these patients.[Table tbl3b]

**Figure 2:**
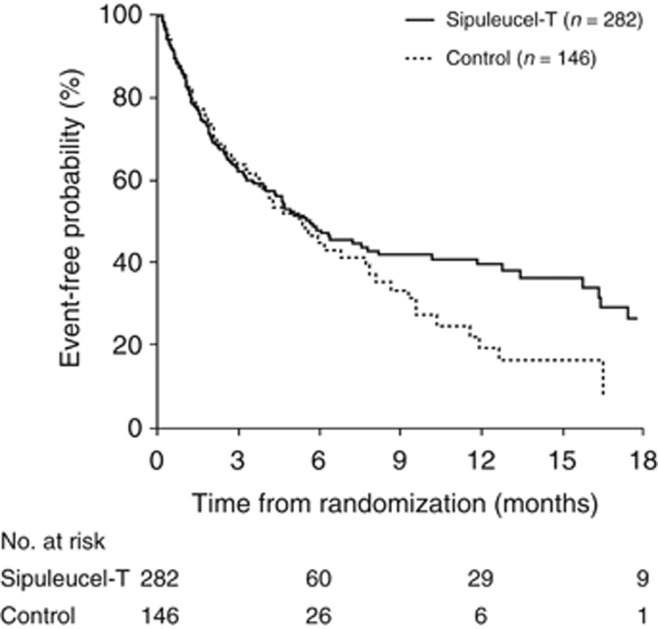
Kaplan–Meier analysis of time to disease-related pain in the pooled data set (intent-to-treat population).

TDRP in the pooled analysis was not significantly delayed with sipuleucel-T versus control (HR=0.819; 95% CI: 0.616–1.089; *P=*0.170) (Table 4a). Median estimated TDRP was 5.6 months (95% CI: 4.3–7.8) with sipuleucel-T versus 5.3 months (95% CI: 3.7–7.7) with control. However, the Kaplan–Meier curves of TDRP begin to diverge ∼6 months following randomization (Figure 2). At 12 months, 39.3% of sipuleucel-T and 18.9% of control patients were estimated as pain-free. There was directional consistency between D9901 and IMPACT, but not D9902A (Table 4a). However, the sample size was smaller in D9902A as enrollment was discontinued early and several baseline factors favored the control arm, including PSA, alkaline phosphatase, LDH and the number of bone metastases.^5^

A stepwise Cox regression model identified the following factors as prognostic for earlier TDRP: higher PSA (HR=1.264; 95% CI: 1.133–1.409; *P*<0.001); higher alkaline phosphatase (HR=1.486; 95% CI: 1.208–1.828; *P*<0.001); younger age (per year decrease) (HR=1.024; 95% CI: 1.007–1.041; *P=*0.005); bisphosphonate use (HR=1.600; 95% CI: 1.171–2.188; *P=*0.003); and prior primary radiotherapy (HR=1.596; 95% CI: 1.199–2.126; *P=*0.001). When adjusting for these factors, results were comparable (HR=0.804; 95% CI: 0.602–1.076; *P=*0.142) (Table 4a).

In IMPACT, where TDRP was recorded regardless of PD status, longer TDRP was associated with longer time to PD and OS (both *P*<0.001). Median TDRP in IMPACT was 4 months. In patients with TDRP ⩽4 versus >4 months, respectively, median time to PD was 10.3 versus 34.7 weeks, and median OS was 22.3 versus 37.0 months.

### TFOA

Table 3b provides a summary of OAU and PD status for all patients (*n*=737). OAU for reasons other than cancer-associated pain (for example, meperidine use for rigors on infusion day) was excluded and is summarized in Table 5. Most patients experienced PD, with 419/488 (85.9%) sipuleucel-T and 216/249 (86.7%) control patients progressing; OAU occurred in 153/488 (31.4%) and 87/249 (34.9%) patients, respectively.

**Figure 3:**
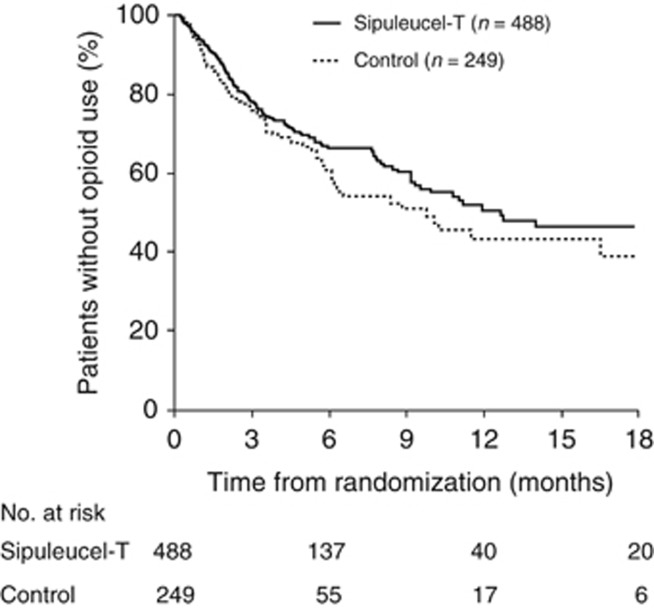
Kaplan–Meier analysis of time to first use of opioid analgesics.

TFOA was significantly delayed for sipuleucel-T versus control (HR=0.755; 95% CI: 0.579–0.985; *P=*0.038) (Table 4b). Median TFOA was 12.6 months (95% CI: 9.3—not estimable) with sipuleucel-T versus 9.7 months (95% CI: 6.0—not estimable) with control. The censoring rate was high (335/488 (68.6%) and 162/249 (65.1%) patients, respectively). Kaplan–Meier estimates of being opioid-free at 12 months were 50.6% for sipuleucel-T compared with 43.1% for control, and curve separation began at 6 months (Figure 3). TFOA results for D9901 and IMPACT, but not D9902A, showed directional consistency (Table 4b) similar to the TDRP analysis.^5^

Significant baseline predictors of shorter TFOA were: higher PSA, alkaline phosphatase and LDH; younger age; higher number of bone metastases; GS ⩾8; ECOG PS 1; higher weight; and prior primary radiotherapy. HR adjusted for these predictors of TFOA was 0.754 (95% CI: 0.571–0.995; *P=*0.046) (Table 4b).

## Discussion

Approximately 90% of patients who die of prostate cancer have bone metastases,^9^ and many experience associated bone pain and receive opioid analgesics.^10^ In addition to its obvious impact on quality of life, pain is an important prognostic factor in mCRPC. A retrospective study of 599 mCRPC patients showed a statistically significant impact of pain on survival time.^1^ Similarly, clinically significant pain was one of the most important independent prognostic factors for OS (HR=1.48; *P*<0.0001) in the pivotal docetaxel TAX327 study of 1006 mCRPC patients.^2^

This analysis of pooled data from three randomized trials demonstrated a significant difference in TFOA for patients treated with sipuleucel-T versus control, but no significant difference in TDRP. Immunotherapies may show a delayed antitumor effect,^11^ which is supported by the divergence of the TFOA and TDRP Kaplan–Meier curves after 6 months and the approximately two-fold increase in patients remaining DRP free at 12 months with sipuleucel-T versus control. Objective PD tended to predate DRP, which in turn preceded OAU (for sipuleucel-T and control, respectively: in IMPACT, median time to PD was 3.7 and 3.6 months;^6^ in this pooled analysis, median TDRP was 5.6 and 5.3 months, and median TFOA was 12.6 and 9.7 months). Given the suggested delayed effect of immunotherapy, the relative kinetics of these end points may explain the increasingly greater treatment effects observed on PD, TDRP and TFOA, successively.

In stepwise logistic regression analyses, several baseline factors were prognostic for earlier TDRP or TFOA. The association of higher PSA, alkaline phosphatase, LDH, number of bone metastases and ECOG PS with shorter TDRP and/or TFOA is consistent with these being baseline markers of more advanced disease. Although the shorter TDRP in patients receiving bisphosphonates seems counterintuitive, bisphosphonate treatment is more common in patients with a higher disease burden. Androgen deprivation therapy treatment is associated with a high risk of clinical fractures so current NCCN guidelines recommend that patients receiving androgen deprivation therapy should also receive bisphosphonates (denosumab, zoledronic acid, or alendronate) when there is an absolute fracture risk.^12^ Higher GS as an indicator for earlier TFOA is consistent with GS being a marker for more aggressive disease.^2, 8^ There is no immediate explanation for the association of younger age or primary radiotherapy with shorter TDRP or TFOA. Importantly, the trend towards delayed TDRP and significantly delayed TFOA with sipuleucel-T versus control were maintained after adjusting for these factors, suggesting that any differences in baseline prognostic factors would not explain the TDRP or TFOA findings.

The delayed antitumor effect of sipuleucel-T may account for the greater treatment effect on later events, such as OAU (TFOA), relative to earlier events, such as DRP (TDRP), or even earlier events such as PD (time to PD). However, the lack of statistical significance versus control for TDRP could also be due to the large amount of censoring (50.5% of patients) and the relatively small sample size (212 observed pain events; >700 pain events would be required to provide 80% power (α=0.05) to detect a true HR of 0.80).

Two randomized phase III studies (NCT01057810 and NCT00861614) are currently evaluating ipilimumab versus placebo for CRPC using pain-related secondary end points. Furthermore, confirmed pain response is the primary outcome (data anticipated in Q3 2014) in the randomized COMET-2 phase III study (NCT01522443) that is evaluating cabozantinib versus mitoxantrone plus prednisone in men with previously treated symptomatic CRPC.

This analysis has several limitations, most notably its *post hoc* nature. For example, while the medical review that was used to exclude OAU unrelated to cancer pain was blinded, the criteria were identified *post hoc* and may have led to differential exclusion favoring one arm over the other. Other limitations are the high degree of censoring and the fact that, individually, each study was not adequately powered for TDRP or TFOA analysis, requiring data pooling.

Nevertheless, this analysis has demonstrated a significant delay in TFOA across the pooled population, irrespective of baseline symptomatic status. The TFOA findings and trend in TDRP are consistent with the delayed antitumor effect reported with immunotherapy in this patient population, and suggest that further study of clinical end points proximate to OS (such as TDRP and TFOA) is warranted.

## Figures and Tables

**Table 1:** Study designs of D9901, D9902A, and IMPACT

*Study design element or end point*	*D9901 and D9902A*	*IMPACT*	
	*(Sipuleucel-T,* n*=147; control,* n*=78)*	*Pre-amendment (sipuleucel-T,* n*=135; control,* n*=68)*	*Post-amendment (sipuleucel-T,* n*=206; control,* n*=103)*
Eligibility criteria	Asymptomatic All Gleason scores	Asymptomatic Gleason score ⩽7	Asymptomatic/minimally symptomatic All Gleason scores
Overall survival	Prespecified analysis	Secondary end point	Primary end point
Time to progression	Primary end point	Co-primary end point	Secondary end point
Time to disease-related pain (TDRP)	Secondary end point	Co-primary end point	End point removed; data collection discontinued
Time to first use of opioid analgesics (TFOA)	Data collected	Secondary end point	End point removed; data collection continued

**Table 2:** Summary of patient demographics and baseline characteristics

*Baseline patient demographic or characteristic*	*TDRP analysis population*			*TFOA analysis population*		
	*Sipuleucel-T (*n*=282)*	*Control (*n*=146)*	*Total (*n*=428)*	*Sipuleucel-T (*n*=488)*	*Control (*n*=249)*	*Total (*n*=737)*
Median age, years (minimum, maximum)	72 (47, 89)	70 (45, 87)	71 (45, 89)	72 (47, 91)	71 (40, 89)	71 (40, 91)
Median weight, kg (minimum, maximum)	88 (55, 159)	86 (60, 136)	87 (55, 159)	88 (53, 175)	86 (60, 136)	87 (53, 175)
Caucasian, *n* (%)	261 (92.6)	136 (93.2)	397 (92.8)	437 (89.5)	229 (92.0)	666 (90.4)
ECOG PS 0, *n* (%)	236 (83.7)	117 (80.1)	353 (82.5)	393 (80.5)	199 (79.9)	592 (80.3)
						
*Gleason score,* n *(%)*						
⩽6	53 (18.8)	23 (15.8)	76 (17.8)	74 (15.2)	31 (12.4)	105 (14.2)
7	176 (62.4)	87 (59.6)	263 (61.4)	277 (56.8)	140 (56.2)	417 (56.6)
⩾8	52 (18.4)	36 (24.7)	88 (20.6)	136 (27.9)	77 (30.9)	213 (28.9)
Missing	1 (0.4)	0	1 (0.2)	1 (0.2)	1 (0.4)	2 (0.3)
						
*Number of bone metastases, n (%)*						
0–5	125 (44.3)	72 (49.3)	197 (46.0)	206 (42.2)	112 (45.0)	318 (43.1)
6–10	40 (14.2)	26 (17.8)	66 (15.4)	67 (13.7)	39 (15.7)	106 (14.4)
>10	113 (40.1)	47 (32.2)	160 (37.4)	211 (43.2)	97 (39.0)	308 (41.8)
Missing	4 (1.4)	1 (0.7)	5 (1.2)	4 (0.8)	1 (0.4)	5 (0.7)
						
Soft-tissue disease, *n* (%)	150 (53.6)^a^	92 (64.3)^a^	242 (57.2)	248 (51.0)^a^	152 (61.8)^a^	400 (54.6)
Median serum PSA, ng ml^−1^^b^	44.7	44.5	44.7	51.5	46.6	49.9
Median alkaline phosphatase, U l^−1^^c^	99.0	103.0	100.0	103.0	104.0	103.0
Median LDH, U l^−1^^d^	186.5	184.5	186.0	190.0	187.5	190.0
Median hemoglobin, g dl^−1^^e^	13.0	13.1	13.1	12.9	12.7	12.9
						
*Treatment history,* n *(%)*						
Combined androgen blockade	245 (86.9)	129 (88.4)	374 (87.4)	411 (84.2)	213 (85.5)	624 (84.7)
Orchiectomy	47 (16.7)	24 (16.4)	71 (16.6)	66 (13.5)	28 (11.2)	94 (12.8)
Chemotherapy	28 (9.9)	14 (9.6)	42 (9.8)	77 (15.8)	33 (13.3)	110 (14.9)
Docetaxel	13 (4.6)	7 (4.8)	20 (4.7)	55 (11.3)	22 (8.8)	77 (10.4)
Radical prostatectomy	107 (37.9)	47 (32.2)	154 (36.0)	180 (36.9)	82 (32.9)	262 (35.5)
Radiotherapy (to the prostate/prostate bed)	163 (57.8)^a^	69 (47.3)^a^	232 (54.2)	267 (54.7)	127 (51.0)	394 (53.5)
						
Median time from diagnosis to randomization, years	6.8	7.1	7.0	6.8	6.8	6.8
Current bisphosphonate use, *n* (%)	70 (24.8)	36 (24.7)	106 (24.8)	175 (35.9)	88 (35.3)	263 (35.7)

Abbreviations: ECOG PS, Eastern Cooperative Oncology Group performance status; LDH, lactate dehydrogenase; TDRP, time to disease-related pain; TFOA, time to first use of opioid analgesics.

a *P*<0.05 for comparison of sipuleucel-T and control. Fisher's exact test for categorical variables and Wilcoxon rank-sum test for continuous variables.

b Normal range ⩽2.7–7.2 ng ml^−1^.

c Normal range 31–131 U l^−1^.

d Normal range 53–234 U l^−1^.

e Normal range 12.5–18.1 g dl^−1^.

**Table 3a:** Summary of disease progression and pain status in time to disease-related pain (TDRP) population

*Patient disposition*	*Sipuleucel-T,* n *(%) (*n*=282)*	*Control,* n *(%) (*n*=146)*
Pain and disease progression	125 (44.3)	69 (47.3)
Disease progression only	118 (41.8)	63 (43.2)
Pain only	12 (4.3)	6 (4.1)
No pain or disease progression	27 (9.6)	8 (5.5)

**Table 3b tbl3b:** Summary of disease progression and opioid use in time to first use of opioid analgesics (TFOA) population

*Patient disposition*	*Sipuleucel-T,* n *(%) (*n*=488)*	*Control,* n *(%) (*n*=249)*
Opioid use and disease progression	139 (28.5)	76 (30.5)
Disease progression only	280 (57.4)	140 (56.2)
Opioid use only	14 (2.9)	11 (4.4)
No opioid use or disease progression	55 (11.3)	22 (8.8)

**Table 4a:** Association between sipuleucel-T treatment and time to disease-related pain (TDRP)

*Study*	*Hazard ratio*	*95% confidence interval*	P*-value*
D9901	0.681	0.373–1.246	0.210
D9902A	1.392	0.652–2.973	0.390
IMPACT	0.802	0.560–1.149	0.227
Integrated result	0.819	0.616–1.089	0.170
Adjusted integrated result^a^	0.804	0.602–1.076	0.142

a Adjusted for the significant baseline predictors of shorter TDRP (higher PSA, higher alkaline phosphatase, younger age, bisphosphonate use and prior primary radiotherapy).

**Table 4b:** Association between sipuleucel-T treatment and time to first use of opioid analgesics

*Study*	*Hazard ratio*	*95% confidence interval*	P*-value*
D9901	0.629	0.304–1.303	0.212
D9902A	1.242	0.544–2.833	0.607
IMPACT	0.727	0.536–0.987	0.041
Integrated result	0.755	0.579–0.985	0.038
Adjusted integrated result^a^	0.754	0.571–0.995	0.046

a Adjusted for the significant baseline predictors of shorter time to disease-related pain (higher PSA, higher alkaline phosphatase, higher lactate dehydrogenase, younger age, higher number of bone metastases, Gleason score ⩾8, Eastern Cooperative Oncology Group performance status 1, higher weight, prior radiotherapy).

**Table 5:** Opioid use excluded from analysis

*Reason for excluding opioid use*^a^	*Sipuleucel-T,* n *(%) (*n*=488)*	*Control,* n *(%) (*n*=249)*	*Total,* n *(%) (*n*=737)*
Any excluded opioid	163 (33.4)	34 (13.7)	197 (26.7)
Rigors due to infusion^b^	116 (23.8)	6 (2.4)	122 (16.6)
Procedure^c^	26 (5.3)	16 (6.4)	42 (5.7)
Short duration^d^	38 (7.8)	12 (4.8)	50 (6.8)
Other^e^	9 (1.8)	4 (1.6)	13 (1.8)

a Patients with multiple excluded opioids are counted once for each reported category.

b Typically rigors and/or chills on the day of an infusion, treated with 1 day of meperidine.

c For example, anesthesia or procedure-related pain.

d Opioids given for 1 or 2 days for reasons other than above (e.g., pain, shortness of breath, respiratory distress).

e Opioids given for >2 days for reasons clearly not due to cancer-related pain (e.g., cough, infection, pain due to injury or accident).

## Notes

Dr Small is an Exelixis Advisory Committee Member, consultant to Dendreon at Advisory Board and Steering Committee meetings, and is a Principal Investigator on a trial involving Sipuleucel-T; Dr Higano is a consultant to Dendreon at Advisory Board and Steering Committee Meetings, and is a Principal Investigator on a clinical trial involving sipuleucel-T; Dr Kantoff is a consultant to Dendreon; Dr Whitmore and Dr Frohlich are Dendreon employees and own Dendreon stock; Dr Petrylak is a consultant to Dendreon and has received grant support from Dendreon.
